# The predictive value of a body shape index as a novel obesity metric for cancer risk: a systematic review and meta-analysis

**DOI:** 10.3389/fnut.2025.1667466

**Published:** 2025-09-24

**Authors:** Hui Liu, Hongjia Fu, Zengyu Wang, Zhicui Yao, Qi Wang, Aixia Sui, Yuan Meng, Xin Xu, Yongqing Shen, Wei Liu

**Affiliations:** ^1^Faculty of Nursing, Hebei University of Chinese Medicine, Shijiazhuang, China; ^2^Sixth Department of Oncology, Hebei Provincial People’s Hospital, Shijiazhuang, China; ^3^College of Clinical Medicine, Jiamusi University, Jiamusi, China; ^4^Hebei Provincial People’s Hospital, Shijiazhuang, China

**Keywords:** a body shape index, cancer risk prediction, colorectal cancer, obesity metrics, meta-analysis

## Abstract

**Background:**

The A Body Shape Index (ABSI), a metric assessing visceral adiposity distribution by integrating height, weight, and waist circumference (WC), remains a subject of debate regarding its predictive value for diverse cancer risks. This study aims to assess the predictive capacity of ABSI for cancer utilizing systematic review and meta-analysis methodologies, contrasting its performance against conventional anthropometric indices such as body mass index (BMI) and WC.

**Methods:**

A comprehensive search of PubMed, Web of Science, and Embase databases was performed from inception through April 27, 2025, to identify observational studies examining associations between ABSI and cancer. Random-effects or fixed-effects models were employed to calculate pooled hazard ratio (HR), odds ratio (OR), and area under the receiver operating characteristic curve (AUC) metrics with corresponding 95% confidence interval (CI), selected according to heterogeneity thresholds. Furthermore, heterogeneity and publication bias were also assessed.

**Results:**

This study included 10 studies (7 cohort studies and 3 cross-sectional studies) with a total sample size of 1,520,762 participants. Results indicated that each one-standard-deviation increase in ABSI was associated with an 8% increase in overall colorectal cancer (CRC) risk, with a significant 13% increase in men and a 6% increase in women. In terms of predictive efficacy, ABSI (pooled AUC = 0.66) outperformed other anthropometric indicators, though in men, each one-standard-deviation increase in ABSI was associated with a significantly lower risk increase by 7% compared to WC. Additionally, no significant association was found between ABSI and the risk of prostate or breast cancer.

**Conclusion:**

ABSI demonstrates remarkable specificity for specific cancer types in cancer risk prediction. It independently predicts CRC risk, particularly in identifying high-risk male populations with central adiposity, serving as a beneficial supplementary tool to WC rather than a substitute. Available evidence does not support the routine application of ABSI for predicting prostate or breast cancer risks. Future studies with larger and more diverse samples are necessary to further verify the effectiveness of ABSI and strengthen its evidential basis.

**Systematic review registration:**

https://www.crd.york.ac.uk/PROSPERO/view/CRD420251047230.

## Introduction

Globally, obesity prevalence is escalating at an alarming pace, constituting a critical public health challenge. According to the World Obesity Atlas 2025, 41% of Chinese adults exhibit elevated body mass index (BMI, ≥25 kg/m^2^), with an obesity prevalence rate of 9%, while 75% of US adults demonstrate elevated BMI alongside a 44% obesity rate (1). Should current trajectories persist, approximately 3 billion adults worldwide—nearly 50% of the global adult population—will be overweight or obese by 2030 (1). Notably, obesity or “excess body fat” has been identified as a risk factor for at least 13 types of cancer—obesity-associated cancers (OACs) (2). As a significant preventable risk factor for cancer, the optimization of clinical assessment and risk prediction tools for obesity is of great importance.

Traditional indices, such as BMI, have long been utilized as straightforward metrics for evaluating obesity. Nevertheless, BMI, as an isolated metric of obesity, possesses considerable limitations. In the realm of cancer, the definition of obesity must extend beyond BMI and employ a multi-index integration approach to assess general and visceral obesity, with the objective of more precisely determining the influence of obesity on cancer outcomes (3). The Lancet Diabetes & Endocrinology Commission recently emphasized that relying exclusively on BMI to define obesity may result in issues like overdiagnosis, and clinical evaluations should include at least one anthropometric measure, such as waist circumference (WC) (4). In recent years, a new obesity index — the A Body Shape Index (ABSI), calculated as ABSI = WC/(BMI^⅔^ * height^½^) — has attracted increasing research attention by integrating measurements of height, weight, and WC, avoiding collinearity with BMI, and enabling a more accurate reflection of visceral fat proportion (5). This attribute enables more precise risk categorization among obese individuals and addresses the limitations of depending exclusively on general or central obesity metrics, hence enhancing research into the correlation between obesity and cancer risk (6, 7). Numerous studies have investigated the association between ABSI and the risk of various cancers; however, their findings remain inconsistent. For example, a European prospective cohort study found that abdominal obesity, as measured by ABSI, was not associated with overall breast cancer risk but was inversely associated with postmenopausal breast cancer risk (8). In contrast, a prospective study utilizing UK Biobank data reported a positive association between ABSI and the risk of five specific cancers, including colorectal and breast cancer, as well as overall cancer risk (9). Notably, for colorectal cancer (CRC), further studies suggest a significant association between ABSI and risk in men, whereas the association remains inconsistent in women (10, 11). These inconsistencies may arise from the heterogeneous role of ABSI across different cancer types and populations, limiting the reliability and generalizability of the existing evidence. Furthermore, ABSI has demonstrated predictive value for a range of diseases (12, 13), indicating its broad utility beyond oncology and providing a rationale for the present study.

Therefore, the purpose of this study is to systematically retrieve and screen relevant literature, synthesize scientific evidence on the predictive value of ABSI, and quantify effect sizes via meta-analysis to explore whether ABSI can independently predict cancer risk beyond traditional obesity indicators (e.g., BMI) and the predictive discrepancies between ABSI and other common anthropometric markers (e.g., WC). This study is expected to provide novel perspectives and empirical evidence for evaluating ABSI in screening cancer-risk populations and offer insights into formulating personalized cancer prevention strategies in clinical practice.

## Materials and methods

This systematic review and meta-analysis followed the 2020 Preferred Reporting Items for Systematic Reviews and Meta-Analyses (PRISMA) guidelines (14). The complete PRISMA 2020 checklist is in Supplementary Table S1. The detailed protocol for this study was registered with the International Prospective Register of Systematic Reviews (PROSPERO) under the registration number CRD420251047230.

### Search strategy

We systematically searched the PubMed, Web of Science, and Embase databases from the inception of each database to April 27, 2025. The search strategy employed a combined approach of Medical Subject Headings (MeSH) and free-text keywords. The core search terms were: (“Cysts” OR “tumour” OR “cancer” OR “Neoplasms”) AND (“A Body Shape Index” OR “ABSI”). Taking PubMed as an example, the specific search formula was as follows: ((“Cysts”[Title/Abstract]) OR (“tumour”[Title/Abstract]) OR (“cancer”[Title/Abstract]) OR (“Neoplasms”[Mesh])) AND ((“A Body Shape Index”[Title/Abstract]) OR (“ABSI”[Title/Abstract])). After removing duplicates, we screened articles by title and abstract. We then retrieved full-text studies for assessment against the inclusion criteria. The initial search and study selection were done by two researchers (F.H.J. and L.H.) independently. Disagreements were resolved by consensus with a third independent researcher (L.W.). We identified potentially relevant articles for full-text review. Our search algorithm excluded terms related to other anthropometric indicators since studies not evaluating ABSI were excluded based on the criteria. When studies met the criteria and included other anthropometric indicators (e.g., BMI), we extracted relevant quantitative estimates to compare ABSI’s predictive ability for cancer with other indicators within the sample.

### Study eligibility criteria

Studies were included if they met all these criteria: (1) Outcome: Cancer. (2) Anthropometric indicator: ABSI. (3) Subjects: Adults aged ≥18. (4) Objective: To assess the ABSI-cancer risk relationship. (5) Design: Cohort or cross-sectional study. (6) Language: Written in English. Studies were excluded if they met any of these criteria: (1) No reported health outcomes related to ABSI. (2) Letters, editorials, commentaries, study/review protocols, or review articles. (3) Full text unavailable. (4) Studies with data formats or models that were not comparable to other studies and could not be converted for pooled analysis.

### Data extraction

We used a standardized data extraction form for each included study. Variables collected included methodology and results: first author, publication year, country, study design, data source, sample size, age range, percentage of women, disease status, follow-up duration, adjusted confounders, statistical methods, and ABSI measurement (self-reported or objective). Outcome variables included odds ratio (OR), hazard ratio (HR), area under the receiver operating characteristic curve (AUC), and estimated predictability of anthropometric indices such as ABSI, BMI, WC, WHtR, etc.

### Quality assessment

Study quality was assessed using the NIH quality assessment tool for observational cohort and cross-sectional studies (15). This tool scores studies based on 14 criteria. Each criterion is scored 1 for “Yes” and 0 for “No,” “Not Applicable,” “Not Reported,” or “Unclear.” The total score is the sum of all criterion scores, ranging from 0 to 14. Two assessors (F.H.J. and L.H.) independently evaluated the quality of each included study. Any disagreements were resolved by a third researcher (L.W.). All quality assessment results of the included studies are detailed in Supplementary Table S4. Study quality assessment reflects the strength of scientific evidence but does not affect inclusion.

### Statistical analysis

Statistical analyses were performed using STATA version 18.0. To evaluate aggregate effect sizes for ABSI’s cancer predictive capacity, meta-analyses were conducted only when ≥2 studies employed identical outcome measures. Predictive differences between ABSI and comparator indices (BMI, WHR, WHtR, WC, etc.) were assessed using studies reporting multiple metrics. Effect sizes were standardized to per 1-standard-deviation (SD) increment for each anthropometric indicator. For studies that reported the HR or OR per specific amount of increase in anthropometric measures, we first calculated the logarithm of the HR (log(HR)) or the logarithm of the OR (log(OR)), multiplied it by the study-specific standard deviation of the anthropometric measure, then exponentiated the result of this calculation to ultimately obtain the HR or OR corresponding to an increment of one standard deviation in the level of the anthropometric measure. For pooling effect sizes, in studies reporting ORs where the incidence of the studied cancers (CRC and prostate cancer) was low (< 10%) (16), ORs provide a reliable approximation of HRs under this condition. These ORs were therefore pooled, and the results are ultimately presented as ORs (17, 18). When comparing ABSI’s predictive ability with other indices, we first calculated the effect size difference between ABSI and other indicators in each study, then estimated the pooled predictive difference. Heterogeneity was assessed using the I^2^ statistic and Cochran’s Q test. In accordance with Cochrane recommendations, this study used the Higgins I^2^ statistic to quantify heterogeneity among included studies. I^2^ values of 0, 25, 50, and 75% typically correspond to negligible, low, moderate, and high heterogeneity, respectively, with an I^2^ > 75% considered indicative of significant heterogeneity (19). When pooling effect sizes, a fixed-effects model was used for low to moderate heterogeneity (I^2^ ≤ 75%), whereas a random-effects model was applied for significant heterogeneity (I^2^ > 75%) (20). The random-effect model assumes that true effects may vary due to between-study heterogeneity. A leave-one-out approach was used to conduct sensitivity analyses for each cancer type, aiming to assess the robustness of the pooled results. Publication bias was assessed using Begg’s and Egger’s tests, with Egger’s test being a commonly used statistical method for evaluating funnel plot asymmetry (21). In all analyses, *p* < 0.05 was considered statistically significant.

## Results

### Study selection

**Figure 1:**
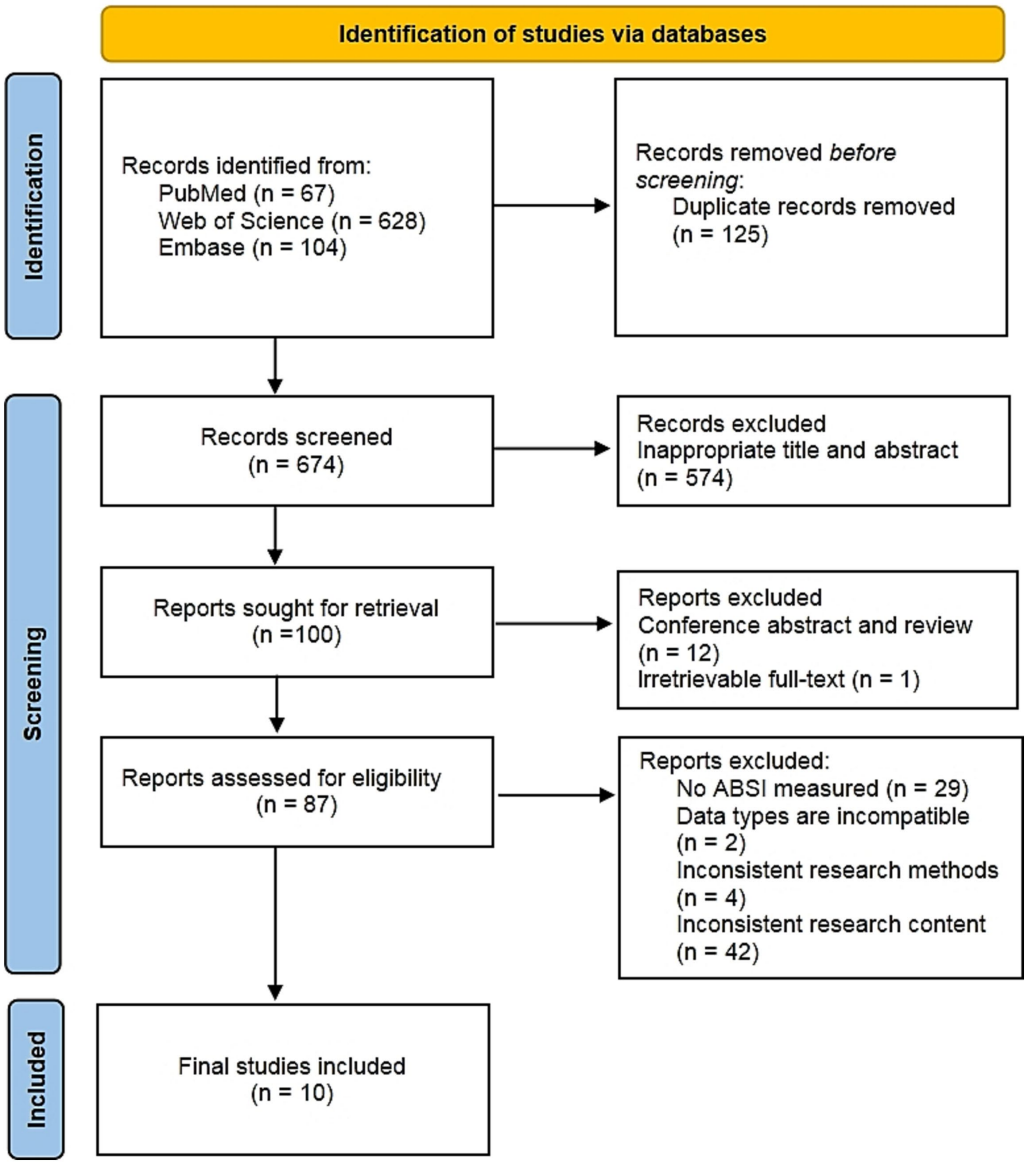
Study selection flow chart.

The initial search identified 799 records, from which 125 duplicates were eliminated. Following title and abstract screening of the remaining 674 records, 574 were excluded. Subsequently, 12 conference abstracts and review articles were removed, along with one article unavailable in full text. Full-text assessment of the remaining 87 articles resulted in the exclusion of 77 publications failing to meet inclusion criteria. Exclusions comprised: 29 articles lacking assessment of ABSI; 2 studies with incompatible data types; 4 utilizing methodologically inconsistent approaches; and 42 publications containing irrelevant content. Ultimately, 10 qualifying studies were included (6, 8–10, 22–27). The full study selection process is shown in the PRISMA flowchart in Figure 1.

### Basic characteristics of the selected studies

The basic characteristics of the 10 included studies are summarized in Supplementary Table S2. These investigations spanned ten distinct nations: the United States (*n* = 3), United Kingdom (*n* = 4), France (*n* = 1), Netherlands (*n* = 1), Australia (*n* = 1), Poland (*n* = 1), Spain (*n* = 1), Sweden (*n* = 2), Italy (*n* = 1), and Denmark (*n* = 1). Study designs included 3 cross-sectional and 7 cohort studies, including 1 multicenter prospective cohort study [by Christakoudi et al. (26), covering multiple European national cohorts] and 6 single-center prospective cohort studies. The total sample size was 1,520,762 participants, with a median of 68,957 (range:11,013 to 442,610). All studies used nationally representative health survey data and calculated ABSI from objective measurements of height, weight, and WC. Study populations were mainly general populations (8 studies), with 1 focusing on patients with metabolic syndrome and 1 on patients with diabetes and obesity. For the seven cohort studies, follow-up durations spanned 7 to 21.5 years (mean: 12.4 years; median: 10.9 years), with one study reporting gender-specific follow-up periods. Every study implemented covariate adjustment for key confounding variables, including demographic characteristics (age, sex, ethnicity, education level) and health-related factors (smoking status, alcohol consumption, physical activity, dietary patterns, family medical history).

Supplementary Table S3 summarizes the main results and assessed predictive indicators of the 10 included studies. Following systematic literature search and screening, the studies that ultimately met the inclusion criteria focused exclusively on three cancer types: CRC (6 studies), prostate cancer (6 studies), and breast cancer (4 studies). Three studies analyzed all three cancer types, while the remaining seven focused on a single cancer type. All 10 studies assessed ABSI. Additionally, 9 evaluated BMI, 6 evaluated WC, and 4 evaluated WHtR. Six studies also assessed other anthropometric indicators.

### Meta-analysis

#### Prostate cancer

**Figure 2:**
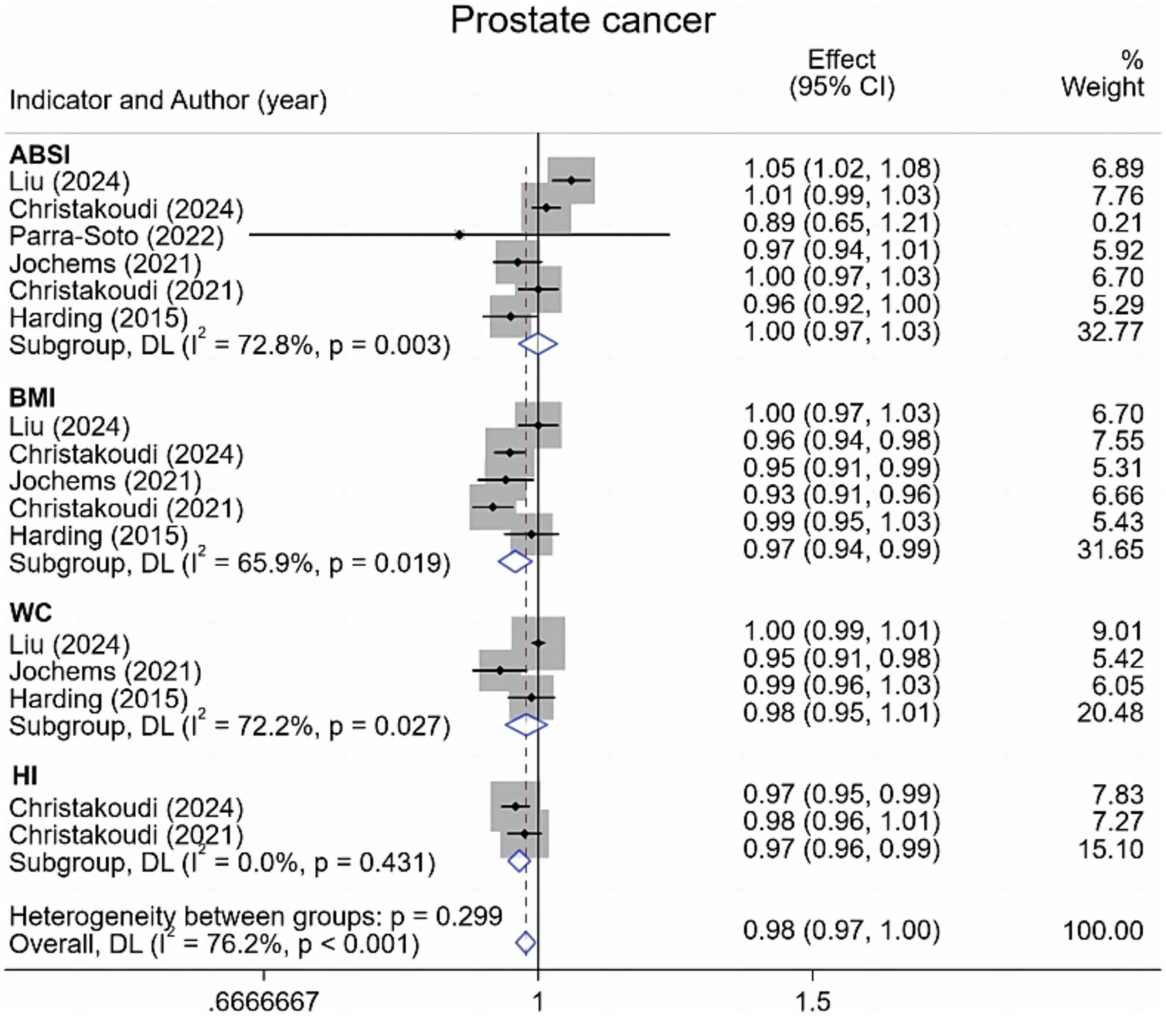
Meta-analysis of the associations between different anthropometric indicators (ABSI, BMI, WC, HI) and prostate cancer risk.

Regarding prostate cancer, this meta-analysis incorporated six studies examining various adiposity metrics. Results (Figure 2) demonstrated no significant association between ABSI or WC and prostate cancer risk. Conversely, each standard deviation (1-SD) increment in BMI corresponded to a 3% reduction in prostate cancer risk [0.97 (95% CI: 0.94–0.99; I^2^ = 65.9%)]. Similarly, a 1-SD increase in hip index (HI) yielded a 3% risk reduction [0.97 (95% CI: 0.96–0.99; I^2^ = 0.0%)].

#### Breast cancer

**Figure 3:**
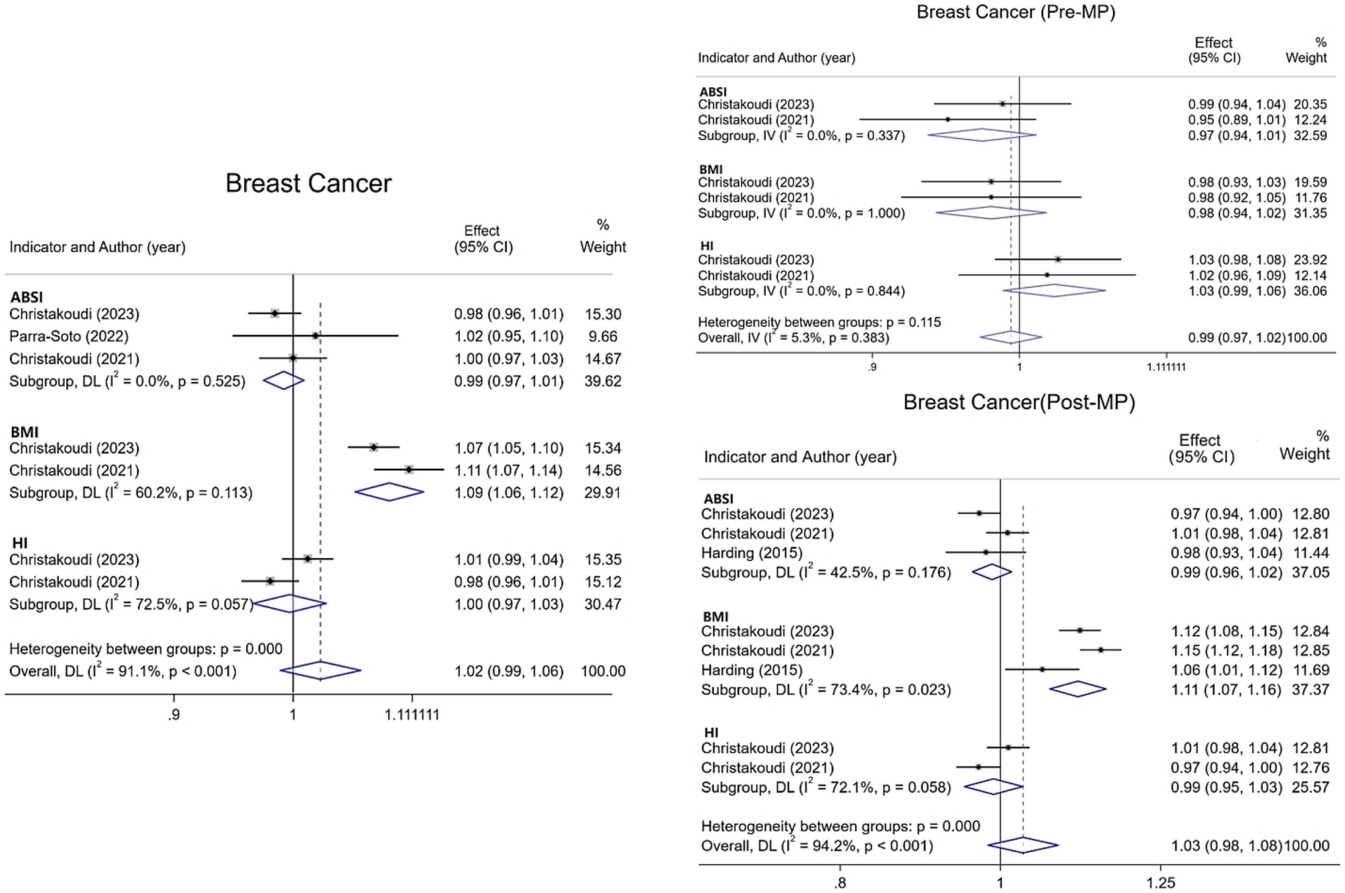
Meta-analysis of the association between different anthropometric indicators (ABSI, BMI, HI) and breast cancer risk in pre- and post-menopausal strata and overall.

Regarding breast cancer, this meta-analysis incorporated four studies examining overall incidence along with premenopausal and postmenopausal subgroups. Results (Figure 3) demonstrate no statistically significant association between ABSI or HI and overall breast cancer risk. However, each 1-SD increase in BMI was associated with a 9% elevation in overall breast cancer risk [1.09 (95% CI: 1.06–1.12; I^2^ = 60.2%)]. For premenopausal breast cancer, neither ABSI, BMI, nor HI exhibited significant associations with risk. Among postmenopausal women, ABSI and HI similarly demonstrated no significant relationship with risk. Conversely, BMI showed an 11% increased risk [1.11 (95% CI: 1.07–1.16; I^2^ = 73.4%)], highlighting how menopausal status significantly modifies BMI’s relationship with breast cancer.

#### Colorectal cancer

**Figure 4:**
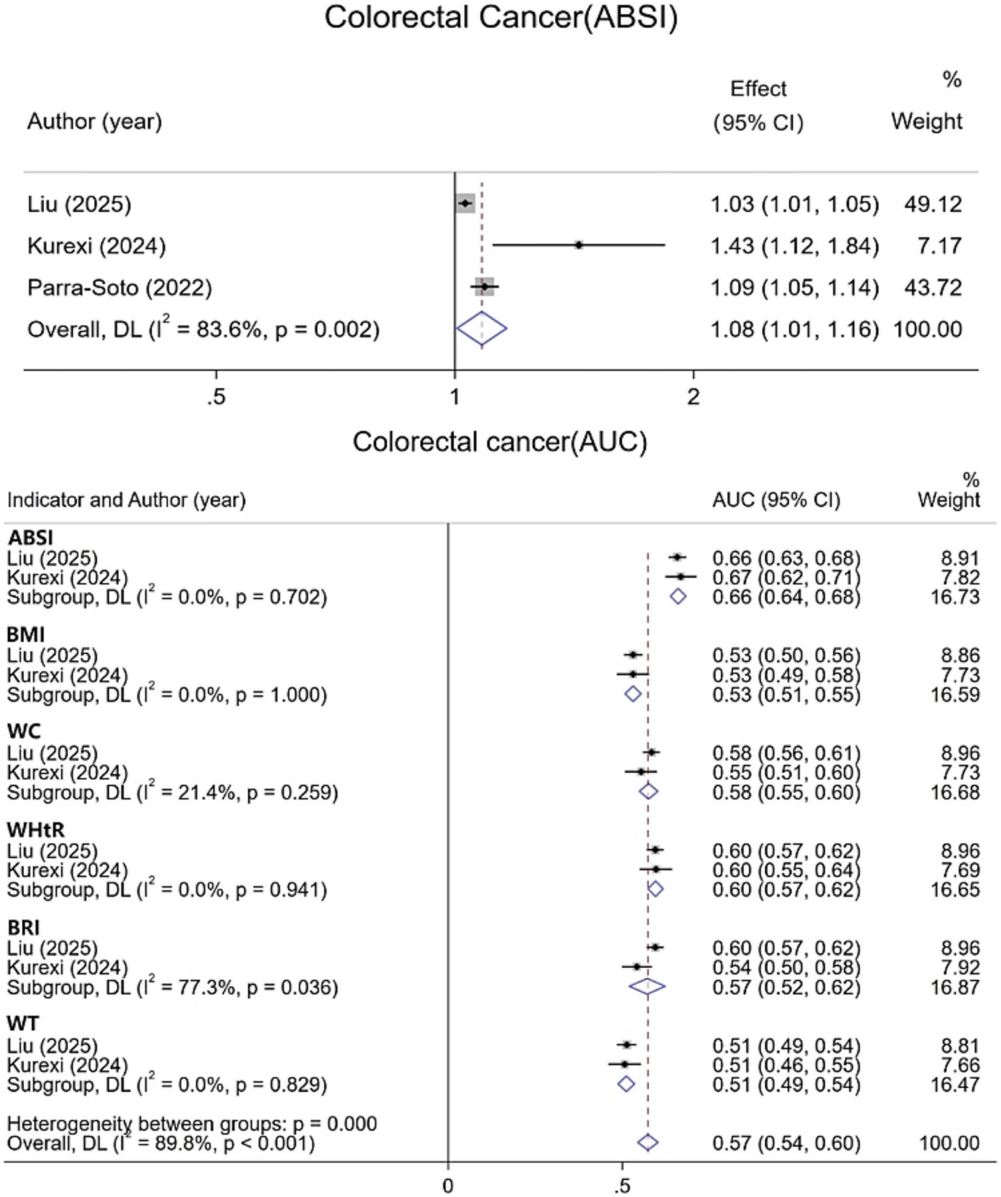
Meta-analysis of ABSI, colorectal cancer risk, and multi-indicator predictive AUC.

**Figure 5:**
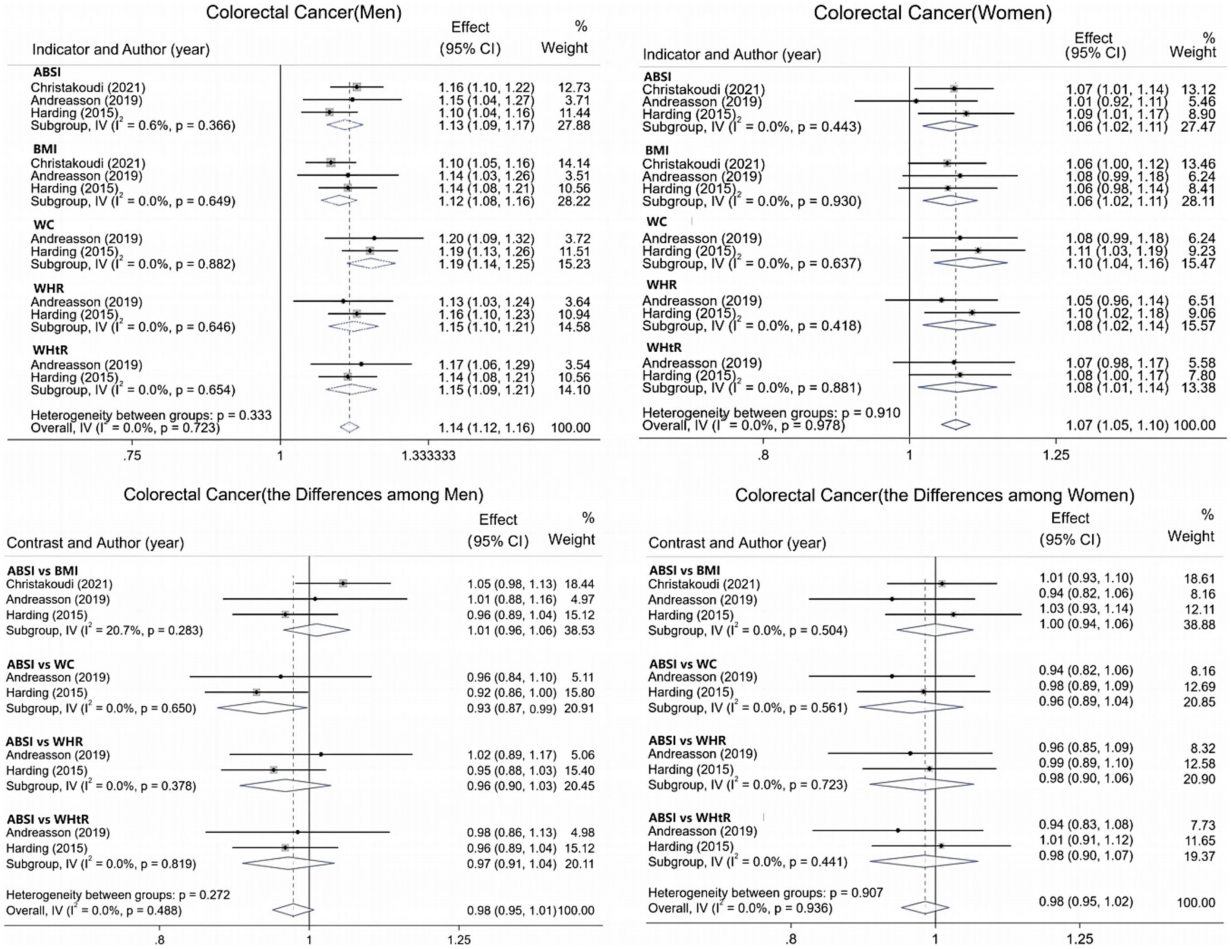
Sex-stratified meta-analysis of anthropometric indicators and colorectal cancer risk.

For CRC, data from six studies were integrated and systematically evaluated by gender stratification to assess the association and predictive ability of various obesity indicators with CRC risk. Results show (Figure 4) that each 1-SD increase in ABSI is associated with an 8% increase in CRC risk [1.08 (95% CI: 1.01–1.16; I^2^ = 83.6%)]. The pooled AUC for ABSI in CRC prediction is 0.66 (95% CI: 0.64–0.68; I^2^ = 0.0%), superior to other indicators. In men (Figure 5), all obesity indicators are significantly and positively correlated with CRC risk (I^2^ ≤ 0.6%). A 1-SD increase in ABSI is linked to a 13% higher risk [1.13 (95% CI: 1.09–1.17; I^2^ = 0.6%)], and a 1-SD increase in WC is associated with a 19% increase [1.19 (95% CI: 1.14–1.25; I^2^ = 0.0%)]. For BMI, WHR, and WHtR, each 1-SD increase is associated with a 12 to 15% risk increase. In women, all indicators are also significantly and positively correlated with CRC risk (I^2^ = 0.0%). For ABSI, a 1-SD increase is associated with a 6% higher risk [1.06 (95% CI: 1.02–1.11)], and for WC, a 1-SD increase is linked to a 10% increase [1.10 (95% CI: 1.04–1.16)]. For BMI, WHR, and WHtR, each 1-SD increase is associated with a 6 to 8% risk increase. Additionally, gender-specific evaluations assessed the predictive differences between ABSI and other obesity indicators. In men, no significant differences were found between ABSI and BMI, WHR, or WHtR. However, the risk increase associated with a 1-SD increase in ABSI was 7% lower than that for WC (95% CI, 0.01–0.13; I^2^ = 0.0%). In women, no significant differences were observed between ABSI and BMI, WC, WHR, or WHtR (*p* > 0.05).

#### Sensitivity and subgroup analysis

To assess the robustness of pooled effect estimates across different cancer types, we performed sensitivity analyses using the leave-one-out method. Results showed that the association results for prostate cancer, breast cancer, and sex-stratified CRC were relatively stable and not unduly influenced by any single study. However, the pooled results for the association between ABSI and CRC in the overall population exhibited poor stability, suggesting that this specific finding requires cautious interpretation. Detailed results of the sensitivity analyses for all cancer types are provided in Supplementary materials. Furthermore, to investigate potential sources of significant heterogeneity (I^2^ = 83.6%) in the association between ABSI and CRC in the overall population, we conducted additional subgroup analyses stratified by sample size, country, and study design (Supplementary Figure S1). The results indicated that heterogeneity remained high and was not significantly mitigated even under stratified conditions. In conjunction with the table of basic characteristics of the three studies included in the CRC meta-analysis, differences in study design, sample size, participants’ disease status, study country, age range, and confounder adjustment are likely the main sources of the significant heterogeneity.

#### Study quality assessment and publication bias

Detailed results of the quality assessment for included studies are summarized in Supplementary Table S4. The mean quality score of the studies was 10.3 out of 14, with a range of 8 to 13. The main sources of bias included: failure to justify the rationale for sample size, inadequate blinding of outcome assessors, and the use of cross-sectional design in 30% of the included studies. These methodological limitations—particularly the inherent challenges in causal inference with cross-sectional designs and the risk of measurement bias—may affect the precision of observational associations and should be considered when interpreting the overall findings of this study, especially those with high heterogeneity. Egger’s test indicated no publication bias (*p* > 0.1).

## Discussion

This systematic review and meta-analysis evaluated the predictive utility of ABSI across various cancers. For CRC, each standard deviation increment in ABSI correlates with an 8% elevation in overall risk (13% in men, 6% in women). ABSI shows a higher pooled AUC for CRC prediction than WC, BMI, etc. Notably, ABSI showed a 7% attenuated risk elevation per 1-SD increase compared to waist circumference in male populations. ABSI is not significantly associated with prostate or breast cancer. Contrastingly, higher BMI correlated positively with breast cancer risk while demonstrating an inverse association with prostate cancer risk. These results highlight the distinct biological implications of different obesity metrics and their varied associations with specific cancer mechanisms.

Comparative analysis of adiposity metrics underscores the imperative for multidimensional obesity assessment in cancer risk stratification. Despite its ubiquity, BMI inherently fails to capture fat distribution patterns, particularly visceral adiposity, a well-established contributor to carcinogenesis (28–30). In our study, ABSI demonstrated the ability to independently predict CRC risk. This not only reinforces the role of central obesity in CRC development but also corroborates findings from multiple large-scale cohort studies (9, 31, 32). Meanwhile, results from a large European cohort demonstrate that ABSI outperforms other abdominal obesity indices in stratifying mortality risk, further solidifying its potential for clinical application (7). Furthermore, both the UK Biobank cohort and a Mendelian randomization study observed that ABSI was significantly associated with the risk of colon and rectal cancer in men, whereas in women, it was only associated with colon cancer risk (11, 22). This sex-specific association aligns with the anatomical site heterogeneity of CRC itself: proximal (right-sided) CRC, which is more common in women, is predominantly located in the ascending colon and cecum and often accompanied by molecular features such as BRAF mutations. In contrast, men are more likely to develop left-sided (distal) CRC, typically found in the descending colon, sigmoid colon, and rectum, which is often characterized by KRAS mutations, among other features (33). The underlying mechanism for this sex and site distribution difference may relate to men’s greater propensity to accumulate visceral fat, whereas women are protected by hormonal levels and unique fat distribution patterns (34–36). Molecular epidemiological studies provide further evidence. Although ABSI shows a consistent and robust association with overall CRC risk, no significant differences were observed in analyses targeting key molecular markers (e.g., BRAF mutations, KRAS mutations) (37). This suggests that central obesity, as reflected by ABSI, does not act through selective pathways for specific molecular subtypes, but rather serves as a “fundamental driver” of CRC initiation and progression via systemic pathological processes such as chronic inflammation, insulin resistance, and intestinal barrier disruption (6, 24). Thus, ABSI can serve as an independent predictor of CRC risk, particularly for identifying high-risk populations associated with visceral fat.

Notably, ABSI was designed to quantify abdominal obesity independent of BMI, and theoretically may more accurately reflect visceral fat accumulation than simple WC (5, 38, 39). However, our results indicate that ABSI underperforms compared to WC in men. This suggests that relying solely on ABSI may not fully capture the biological nuances of CRC risk in men, especially aspects directly related to abdominal obesity. While ABSI might confer theoretical advantages in discriminative accuracy, WC persists as a pragmatically efficient predictor in clinical practice. This conclusion aligns with prior research where ABSI demonstrated no superiority over WC in chronic disease prediction and remains unlikely to supplant traditional obesity metrics for CRC risk assessment (32, 40). Thus, ABSI can complement WC as an auxiliary tool in CRC screening, particularly for men, enabling a more comprehensive assessment of CRC risk.

Additionally, the lack of a significant association between ABSI and prostate or breast cancer is noteworthy, underscoring the complex relationship between obesity indices and cancer risk. Notably, ABSI’s lack of association with prostate cancer contrasts sharply with BMI’s observed inverse relationship. This inverse association with BMI has been consistently observed in multiple large-scale cohort studies, including the European Prospective Investigation into Cancer and Nutrition (EPIC) and the UK Biobank (41, 42). This contradiction may arise from BMI’s inability to distinguish between lean mass (muscle) and fat mass. This limitation contributes to the “obesity paradox,” where higher BMI in men, particularly athletes, often correlates with greater muscle mass, which may partially offset the negative health effects of fat (43, 44). Disparities in screening behaviors may further confound this relationship, as normal-weight men potentially undergo more frequent prostate cancer screening than obese counterparts, potentially delaying diagnosis in the latter group (27, 45). In contrast, ABSI was designed to quantify abdominal fat distribution and may theoretically reflect visceral fat accumulation—a factor linked to increased cancer risk—more accurately than traditional metrics like BMI. However, despite these theoretical advantages, ABSI still showed no significant association with prostate cancer. This highlights the multifaceted pathogenesis of prostate cancer, influenced by androgen metabolism and genetic variability (46, 47), suggesting inherent limitations in ABSI’s predictive capacity that warrant further mechanistic investigation (25). Similarly, ABSI’s null association with breast cancer diverges from BMI’s positive risk correlation observed here, reflecting the distinct biological dimensions captured by different adiposity metrics and their heterogeneous links to site-specific malignancies. Breast cancer risk, particularly for postmenopausal women, has been widely shown to correlate positively with overall obesity, which is typically represented by BMI (48, 49). This suggests that overall obesity burden—better characterized by BMI—rather than central abdominal fat distribution—specifically captured by ABSI—may be the more dominant factor, particularly through systemic hormonal pathways. This finding further explains why the risk of sex hormone-related cancers (such as postmenopausal breast cancer and prostate cancer) is generally unrelated to ABSI (22). BMI, which reflects overall obesity, remains a more effective predictive metric.

For the selection of obesity indicators, the specific pathophysiological mechanisms of the target cancer should be considered. Significant individual differences in genetic background, lifestyle, and metabolic status, along with interactions between lifestyle factors (e.g., diet, exercise, smoking, alcohol) and obesity, can all affect the strength of the association between obesity indicators and cancer risk (50, 51). The complex interplay between obesity and other metabolic abnormalities, such as insulin resistance and chronic inflammation (52), further shows that the obesity-cancer relationship is often due to multiple interacting factors, rather than a simple cause-and-effect link. Thus it can be seen, no single obesity metric can perfectly predict the risk of all types of cancer. The predictive value of obesity indices varies according to their respective capacities to characterize distinct fat distribution patterns and metabolic derangements. Furthermore, ABSI is not a universal metric; its utility is strictly limited to specific cancers (such as CRC) and particular risk assessment contexts, and it should be used in combination with other indicators (especially WC) and risk information. Specifically, in CRC risk assessment for men, WC remains a fundamental indicator, while ABSI can serve as a supplementary tool for individuals at the WC cutoff value or those with normal BMI but elevated WC; in women, the combined use of ABSI and WC improves the precision of risk identification. Their complementary application helps enhance the accuracy and targeting of CRC risk assessment, providing a basis for clinical screening and precise prevention. The findings of this study support ABSI as a valuable complement to WC and BMI, with particular utility in identifying high-risk male populations with a central obesity phenotype. This also aligns with the trend that obesity assessment needs to go beyond a single indicator and move toward multi-dimensional integration (3, 4). However, regarding breast cancer and prostate cancer, current evidence is insufficient to support the routine use of ABSI.

This study comprehensively evaluates ABSI’s role in cancer risk prediction, though several limitations warrant acknowledgment. First, the heterogeneity of included studies affects result comparability. The high heterogeneity observed in the overall analysis of CRC is particularly striking, stemming primarily from inherent differences in study design, population characteristics, and adjusted covariates, among other factors. Thus, the pooled results require cautious interpretation. Cross-sectional studies cannot establish causality, and although cohort studies can observe temporal changes, their results are vulnerable to confounding factors. Secondly, existing evidence, derived primarily from specific regions such as the United States, Europe, and Australia, may have limited global applicability due to geographic and cultural differences, as well as variations in genetic background and lifestyle across populations, which could influence obesity-cancer associations and ABSI’s predictive ability. Third, ABSI calculation relies on WC, height, and weight measurements. Its accuracy is limited by the standardization and measurement error of these basic metrics. Notably, further, due to the range of assessable cancer types being limited by currently published literature, our meta-analysis was restricted to CRC, prostate cancer, and breast cancer, with a limited number of independent studies available for each individual cancer type. Nevertheless, our exploratory evidence from sex-stratified analyses based on low-heterogeneity data supports ABSI as a supplementary tool rather than a replacement for WC in CRC screening. Future research should expand single-cancer studies (at least 5–10 independent cohorts per cancer type) and establish population-specific clinical cutoffs to strengthen ABSI’s evidence base in cancer risk prediction.

## Conclusion

In summary, this systematic review and meta-analysis demonstrates that the selection of obesity metrics for cancer risk prediction should be guided by cancer-specific pathological mechanisms. ABSI can independently predict CRC risk and yields a higher overall AUC than BMI and WC; however, it performs less well than WC in male populations. Thus, ABSI can complement WC—particularly in identifying CRC-susceptible males with central obesity—thereby enhancing the comprehensiveness of risk assessment. Notably, current evidence does not support the routine use of ABSI for predicting prostate or breast cancer risk, and BMI or other mechanism-relevant metrics may be more appropriate for these cancers. Future studies should investigate the predictive mechanisms of ABSI and validate its utility in larger, more ethnically diverse populations. Additionally, efforts are needed to integrate multidimensional obesity metrics, clarify their underlying pathological mechanisms, and thereby refine personalized cancer prevention strategies and risk assessment frameworks.

## Data Availability

The original contributions presented in the study are included in the article/Supplementary material, further inquiries can be directed to the corresponding authors.
